# Locoregional progression and surgical indications in stage IV asymptomatic SI-NETs

**DOI:** 10.1530/ERC-25-0205

**Published:** 2025-08-22

**Authors:** Branislav Klimácek, Tobias Åkerström, Matilda Annebäck, Per Hellman, Staffan Welin, Anders Sundin, Olov Norlén, Peter Stålberg

**Affiliations:** ^1^ Department of Surgical Sciences, Uppsala University, Uppsala, Sweden; ^2^ Department of Medical Sciences, Uppsala University, Uppsala, Sweden

**Keywords:** small intestinal neuroendocrine tumors, tumor growth rate, volumetric measurements, mesenteric lymph node metastasis, non-operative management, symptom onset, stage IV SI-NETs

## Abstract

Small intestinal neuroendocrine tumors are often diagnosed at an advanced stage, with up to 70% of patients presenting with stage IV disease. While some guidelines recommend prophylactic resection of the primary tumor and mesenteric lymph node metastasis in patients without abdominal symptoms at diagnosis to prevent future abdominal complications, the benefit of this approach remains uncertain. This retrospective cohort study included 44 asymptomatic patients with stage IV small intestinal neuroendocrine tumors treated at Uppsala University Hospital between 2014 and 2019. Additional ten symptomatic patients who underwent at least two computed tomography scans before planned surgery were included in the analysis of mesenteric metastasis volume change and tumor growth rate. The primary outcomes were abdominal symptoms development requiring surgical intervention and the assessment of mesenteric metastasis size progression. During a 10-year follow-up, only four initially asymptomatic patients (9%) developed symptoms leading to surgery. Among all 54 patients, the median volume change in mesenteric metastases was −298 mm^3^ (IQR: −2,785–1,294), with no significant difference between baseline and most recent scans (*P* = 0.38). The median interval between scans was 29 months, and the median tumor growth rate was −0.6% per month (IQR: −3.6–1.9%). Similar results were observed in the asymptomatic group. These findings suggest that a non-operative management in stage IV patients without abdominal symptoms is associated with a low incidence of symptom development and limited progression of mesenteric metastases.

## Introduction

Small intestine neuroendocrine tumors (SI-NETs) represent the most commonly diagnosed malignancy affecting the small intestine (1). Primary tumors, despite their small size, often demonstrate early involvement of regional lymph nodes (2, 3, 4). Due to their indolent nature and the absence of early symptoms, stage IV disease, characterized by the presence of distant metastases, is diagnosed in up to 70% of patients (5, 6, 7). The desmoplastic reaction surrounding mesenteric metastasis leads to formation of fibrotic strands, which can affect the small intestine and mesenteric vessels, potentially leading to obstruction, abdominal pain, or ischemia (8, 9, 10). In light of their efficacy to suppress hormonal hypersecretion, relieve symptoms, and inhibit tumor growth (TG) (11, 12, 13), somatostatin analogs (SSAs) are recommended as first-line therapy for unresectable stage IV disease by both the European and the North American neuroendocrine Tumor Societies (ENETS and NANETS, respectively) (14, 15). In the context of surgical management, several studies have suggested a potential survival benefit from resection of the primary tumor and mesenteric lymph node metastasis (MLNM) (16, 17, 18); yet, the findings from other studies have not demonstrated such an advantage (8, 19). Thus, the role of prophylactic surgery in this clinical setting remains controversial. The guidelines recommend resection of the primary tumor and MLNM in patients presenting with abdominal symptoms, and further suggest prophylactic surgery to reduce the risk of future symptom development in asymptomatic patients with unresectable stage IV disease (3, 20). However, the clinical benefit of this preventive approach in the absence of abdominal symptoms remains uncertain. Following the study by Daskalakis *et al.* in (19), surgical resection at our institution has primarily been reserved for patients with abdominal symptoms resulting from the primary tumor and MLNM.

To evaluate this clinical practice, the present study investigates the frequency of symptom onset requiring surgery in initially asymptomatic patients, and seeks to better understand the dynamics of mesenteric metastasis size changes over time.

## Materials and methods

### Study design

This retrospective cohort study included patients diagnosed with stage IV disease SI-NET without symptoms requiring surgery, who were managed by the Department of Surgery or Endocrine Oncology at Uppsala University Hospital between January 1, 2014, and December 31, 2019. Eligible patients were identified through a search of the inpatient registry database at Uppsala University Hospital using the International Classification of Diseases, Tenth Revision (ICD-10) code system, ICD code C17, C17.1 and C17.2. The date of diagnosis was defined as the date of the baseline computed tomography (CT) scan confirming SI-NET disease. Patients who underwent surgery within 3 months of diagnosis were excluded. In addition, symptomatic patients at diagnosis who had follow-up CT scans at least 2 months apart from baseline before planned surgery of primary tumor and MLNM were included in the analysis of mesenteric metastasis size progression.

Written informed opt-out consent was required for any patient who declined to participate in the study. The study was approved by the Swedish Ethical Review Authority (DNR 2022-03848-01).

### Procedures

Patients’ charts were reviewed, and demographic, clinical, histopathological, treatment and radiological data were collected. Primarily, asymptomatic patients at the time of diagnosis were identified to analyze the frequency of symptom onset prompting surgery during follow-up. Second, all patients, regardless of symptom status, who underwent at least two scans more than 2 months apart, were grouped to assess the dynamics of mesenteric metastasis growth.

### Radiological examination for assessment of tumor growth

To evaluate potential TG of MLNM, CT scans were reviewed at baseline and at the most recent follow-up. The CT scans used in this study had slice thickness of 5 mm or less. All imaging studies were assessed by a senior consultant radiologist specializing in neuroendocrine tumors. Tumor growth rate (TGR) was selected as the preferred method for evaluating changes in tumor size (21, 22). Although more complex to calculate than Response Evaluation Criteria in Solid Tumors (RECIST), TGR provides a more detailed and precise assessment of TG dynamics, particularly when volumetric measurements are considered, as was the case in this study. TGR accounts for the time interval between CT scans and is expressed as a percentage, quantifying the rate of change in tumor volume per month (%/m). The mesenteric metastasis shape was approximated as an ellipsoid, with its volume (V) calculated using the formula: $V=\frac{4}{3}.\pi .a.b.c$, where *a*, *b*, and *c* represent the semi-axes of three principal axes derived from measurements in the transverse, coronal and sagittal planes, respectively. For lymph node metastases forming conglomerates, the total volume of the entire cluster was measured as a single entity. Assuming a roughly exponential TG, the relationship between tumor volumes at two time points, baseline (V1) and follow-up (V2) was modeled as: $V2\;=\;V_{1}e^{\text{gt}}$, where t represents the time interval (in months) between the two measurements and g is the TGR constant. This gives TG estimator as: $\text{TG}\;=\;\frac{3}{t}\log\left(\frac{V_{2}}{V_{1}}\right)=\frac{3}{t}\log\left(\frac{D_{2}}{D_{1}}\right)$. The TGR was subsequently derived as the percentage change in tumor volume per month using the formula: $\text{TGR}\;=\;100\left(e^{\text{TG}}-1\right)$ (21).

In addition, the location of mesenteric metastasis was assessed on baseline CT scans using the classification system proposed by Deguelte *et al.* (23), which correlates metastasis location to the superior mesenteric artery and its branches.

### Outcomes

The primary outcomes were the frequency of abdominal symptom development during follow-up in asymptomatic patients at diagnosis, necessitating surgical intervention, and the evaluation of progression in mesenteric metastasis size. The secondary outcomes included the influence of clinical variables on the changes in tumor volume and TGR, the correlation between symptom development and the anatomical location of mesenteric metastasis with respect to the superior mesenteric artery, and overall survival (OS) in patients who were asymptomatic at the time of diagnosis.

## Results

**Figure 1:**
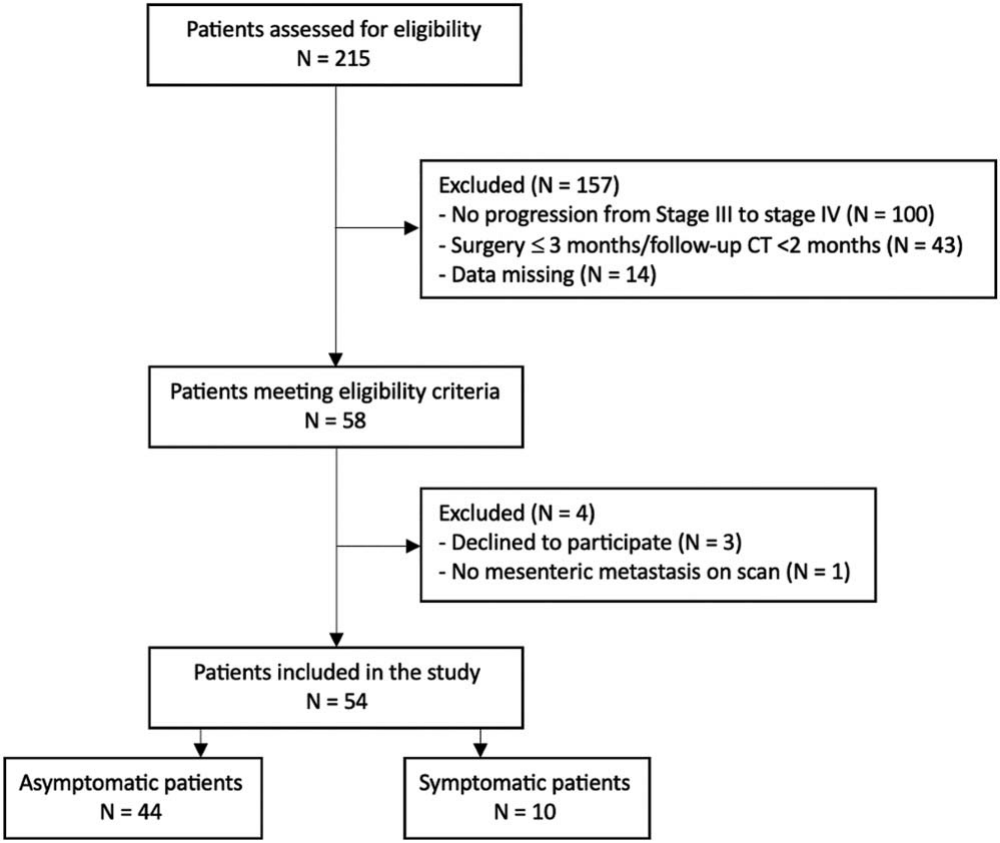
Study flow chart.

Of the 215 patients assessed, 58 met the inclusion criteria. Among them, three declined to participate in the study and one was excluded due to the absence of visible mesenteric metastasis on the CT scan. At baseline, 44 patients were identified as having no symptoms warranting surgical intervention. Ten patients who were symptomatic at the time of diagnosis underwent two or more CT scans before planned surgery, allowing for the assessment of mesenteric metastasis size progression in a total of 54 patients (Fig. 1).

**Figure 2:**
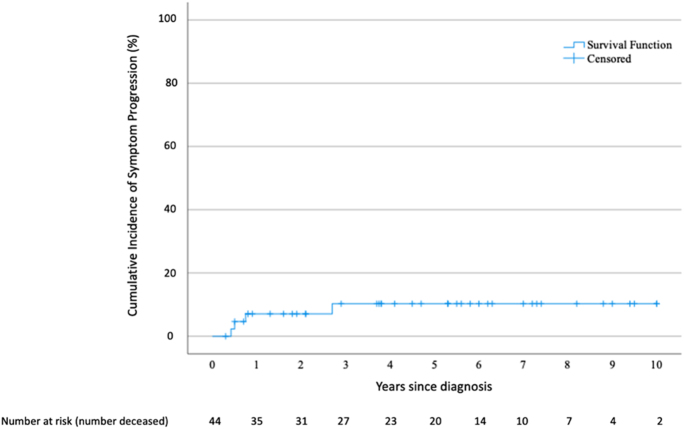
Kaplan−Meier estimates of time to symptom onset in initially asymptomatic patients. A full color version of this figure is available at https://doi.org/10.1530/ERC-25-0205.

During the 10-year follow-up, four patients (9%) required surgery for symptom management (Fig. 2). In one case, only the small bowel containing primary tumor was resected, as the mesenteric metastasis had been considered unresectable upon diagnosis. Indications for surgery included intestinal obstruction (*n* = 2), gastrointestinal bleeding (*n* = 1), and abdominal angina (*n* = 1).

All 54 patients included in the MLNM size progression analysis underwent CT follow-up at least 2 months post-baseline assessment. There were no significant differences between asymptomatic and symptomatic groups in terms of gender, metastatic sites (liver, peritoneal carcinomatosis, and distant metastasis), 5-HIAA levels, Ki-67 index, tumor grade, or the presence of carcinoid heart disease. However, those in the symptomatic subgroup tended to be younger, with a borderline statistical significance (*P* = 0.054) (Table 1). All patients received SSA treatment from the time of diagnosis onward.

**Table 1:** Baseline characteristics.

	Asymptomatic patients *n* = 44	Symptomatic patients *n* = 10	*P* value
Age (years) (IQR)	70 (66–74)	63 (58–70)	0.054
Gender			0.728
Female	21 (48)	6 (60)	
Male	23 (52)	4 (40)	
Deceased	26 (59)	3 (30)	0.26
Metastatic sites			
Liver	43 (98)	10 (100)	1.0
Peritoneal carcinomatosis	5 (11)	2 (20)	0.601
Extra-abdominal	21 (48)	3 (30)	0.483
U 5-HIAA (umol/dL) (IQR)*	335 (100–1,013)	96 (65–240)	0.086
Ki-67%*	4 (1–9)	3.5 (3–6)	0.726
Grade*			0.418
1	18 (41)	2 (20)	
2	24 (55)	8 (80)	
3	1 (2)		
Carcinoid heart disease	13 (30)	1 (10)	0.080

Values are median (Q1;Q3) and number (percent) unless otherwise stated.

5-HIAA (U): urinary 5-hydroxyindoleacetic acid; Ki-67%: values from liver biopsy.

* Missing data: one patient for U 5-HIAA and one for Ki-67% level.

**Figure 3:**
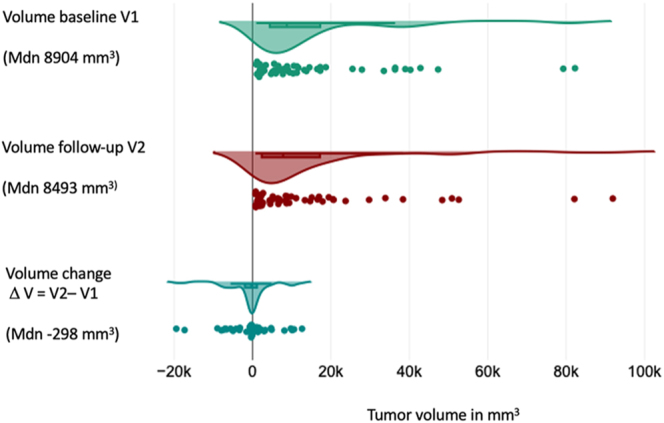
Mesenteric metastasis volume dynamics in asymptomatic and symptomatic patients. A full color version of this figure is available at https://doi.org/10.1530/ERC-25-0205.

**Figure 4:**
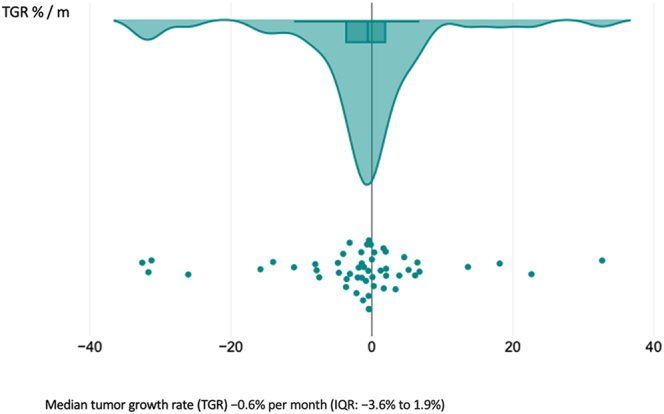
TGR in asymptomatic and symptomatic patients. A full color version of this figure is available at https://doi.org/10.1530/ERC-25-0205.

The overall volume change in the group of asymptomatic and symptomatic patients was −298 mm^3^ (IQR: −2,785–1,294), with no statistically significant difference between the baseline (V1) and most recent (V2) volume measurements (*P* = 0.38) (Fig. 3). The median time interval between CT scans was 29 months (IQR: 7–54), with a TGR of −0.6% per month (IQR: −3.6–1.9%) (Fig. 4).

**Figure 5:**
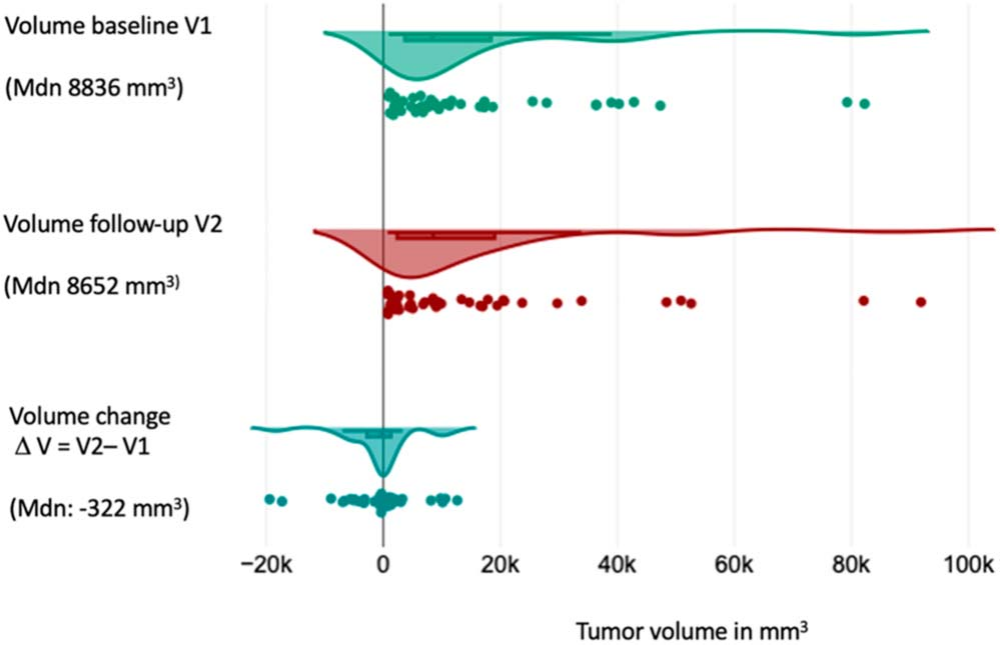
Mesenteric metastasis volume dynamics in asymptomatic patients. A full color version of this figure is available at https://doi.org/10.1530/ERC-25-0205.

**Figure 6:**
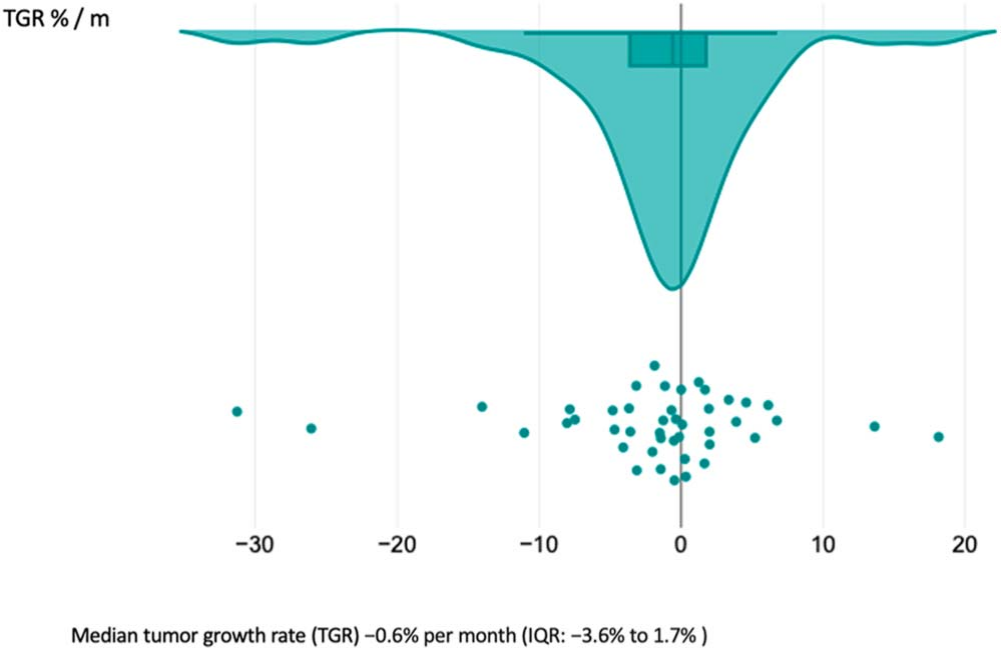
TGR in asymptomatic patients. A full color version of this figure is available at https://doi.org/10.1530/ERC-25-0205.

In the asymptomatic group alone, the median volume change was −322 mm^3^ (IQR: −3,238–1,423). No significant difference was noted between the volume at diagnosis and the most recent radiological assessment (*P* = 0.466) (Fig. 5). The median follow-up duration was 35.5 months (IQR: 13–56), with a TGR of −0.6% per month (IQR: −3.6–1.7%) (Fig. 6).

To evaluate potential factors associated with the progression of MLNM in asymptomatic patients, a logistic regression model was applied. The analysis included age, gender, 5-HIAA levels, Ki-67 index, and tumor grade as covariates, with progression defined by an increase in tumor volume and TGR. None of the variables demonstrated a statistically significant association with MLNM growth. Specifically, age (OR 1.025; 95% CI: 0.948–1.108; *P* = 0.54), gender (female vs male: OR 1.563; 95% CI: 0.372–6.568; *P* = 0.542), 5-HIAA levels (OR 1.000; 95% CI: 0.999–1.001; *P* = 0.763), and Ki-67 index (OR 0.983; 95% CI: 0.782–1.236; *P* = 0.884) were not significantly associated with increased risk (Table 2).

**Table 2:** Logistic regression model fitted for having positive volume change/TGR (*n* = 42).

	Odds ratio	95% confidence interval	*P* value
Constant	0.111	0–30.34	0.442
Age	1.025	0.948–1.108	0.54
Gender			
Male (ref)			
Female	1.563	0.372–6.568	0.542
5HIAA*	1	0.999–1.001	0.763
Ki-67%*	0.983	0.782–1.236	0.884
Grade*			
Grade 1 (ref)			
Grade 2	0.904	0.107–7.664	0.926
Grade 3	0	0 – infinity	0.998

* Two patients were excluded from the analysis due to missing data (one patient from 5HIAA; one patient from Ki-67% and Grade).

During the study period, 16 initially asymptomatic patients underwent peptide receptor radionuclide therapy (PRRT). A trend toward a significant difference in volume change was observed in PRRT-treated patients compared to non-treated (−562 mm^3^ vs −48.5 mm^3^; *P* = 0.06). There was no significant difference in TGR between these groups (−1.2% per month vs 0.04% per month; *P* = 0.313) (Table 3).

**Table 3:** Tumor volume change and TGR between lutetium-treated and non-treated patients.

	PRRT-treated group *n* = 16	Non-treated group *n* = 28	*P* value
Volume change (mm^3^)	−562 (−2,782–8.75)	−48.5 (−454–2,083)	0.06*
TGR (%/m)	−1.2 (−3.3–0.5)	0.04 (−1.54–2)	0.313*

Values are median (Q1;Q3) and number (percent) unless otherwise stated.

PRRT: peptide receptor radionuclide therapy.

* Mann–Whitney U test.

**Figure 7:**
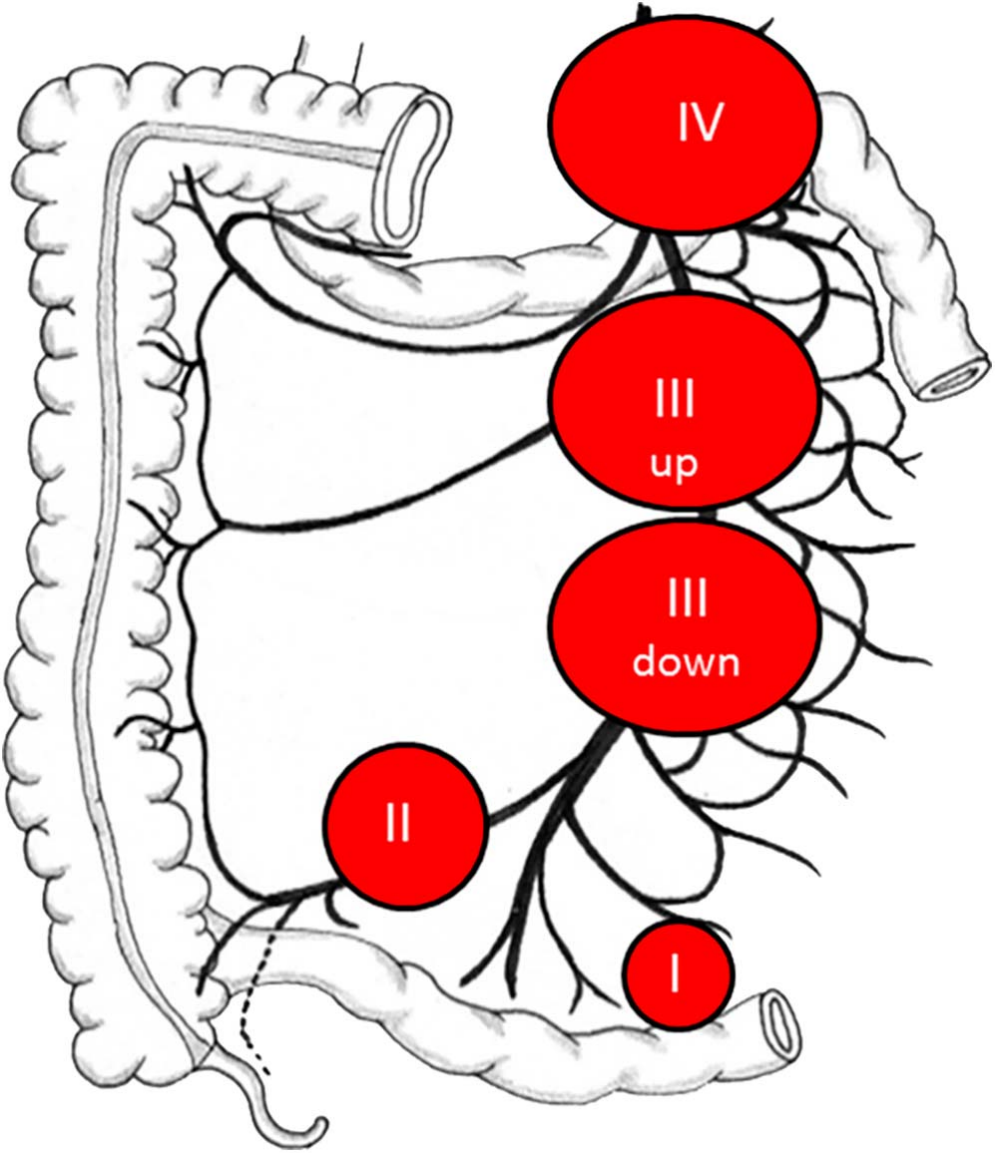
Classification of MLNM location relative to the superior mesenteric artery, as defined by Deguelte *et al.* Reproduced from Deguelte *et al.* (23) (CC BY 4.0). A full color version of this figure is available at https://doi.org/10.1530/ERC-25-0205.

According to the classification by Deguelte *et al.* (23), which categorizes MLNM location based on proximity to the superior mesenteric artery (Fig. 7), MLNM in asymptomatic patients was frequently located in level III down (36%), I (30%), and II (27%), with fewer cases in level III up (5%) and IV (2%). In symptomatic patients at diagnosis, MLNM was most commonly found in level II (50%) and III down (40%), while III up was observed in one case (10%). Among patients who developed symptoms during follow-up, MLNM was distributed across level I, II, III down, and IV, with one case (25%) in each category (Table 4).

**Table 4:** MLNM location relative to the superior mesenteric artery.

	Asymptomatic patients *n* = 44	Symptomatic patients at diagnosis *n* = 10
I	13 (30)	
II	12 (27)	5 (50)
III down	16 (36)	4 (40)
III up	2 (5)	1 (10)
IV	1 (2)	

	Asymptomatic patients *n* = 44	Symptomatic patients during follow-up *n* = 4
I	13 (30)	1 (25)
II	12 (27)	1 (25)
III down	16 (36)	1 (25)
III up	2 (5)	
IV	1 (2)	1 (25)

Values are number (percent); MLNM, mesenteric lymph node metastasis.

**Figure 8:**
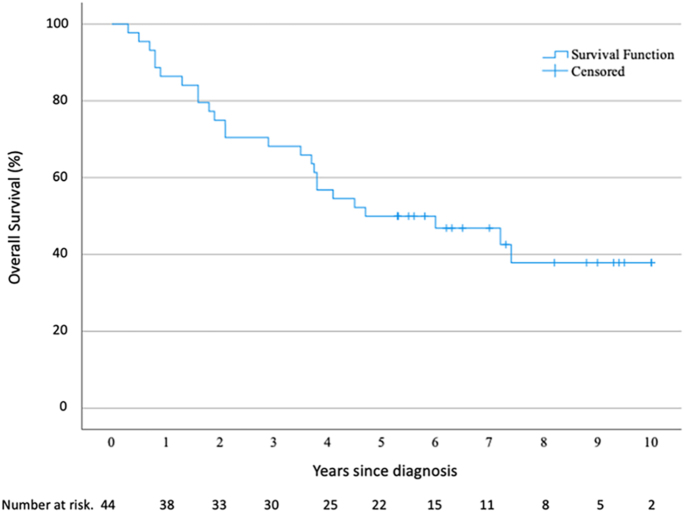
Kaplan−Meier estimates of OS for asymptomatic patients. A full color version of this figure is available at https://doi.org/10.1530/ERC-25-0205.

Among the 44 asymptomatic patients, 25 deaths were recorded over a 10-year follow-up period. The OS rate was estimated at 49.3% (95% CI: 34.4–64.1%) at 5 years and 37.9% (95% CI: 21.3–54.5%) at 10 years (Fig. 8).

## Discussion

This study evaluated the outcomes of a non-operative approach in patients with stage IV SI-NETs without abdominal symptoms at diagnosis. Our current practice of reserving resection for symptomatic patients was implemented following the study by Daskalakis *et al.*, which found no survival advantage from prophylactic resection in the absence of abdominal symptoms (19). Over a 10-year follow-up period, only a small proportion of patients developed symptoms prompting operative intervention, while the mesenteric metastases largely remained stable in size.

Guidelines advocate prophylactic palliative resection of the primary tumor and MLNM, in part to prevent future abdominal complications in initially asymptomatic patients (3, 20). However, the actual likelihood of symptom development in this population remains uncertain. In the study by Daskalakis *et al.*, 58% of initially asymptomatic patients underwent surgery during follow-up, yet only 20 patients (22%) in the cohort had abdominal symptoms explicitly identified as the primary indication for surgery. For the remaining patients, the specific rationale for the operation was not clearly defined, suggesting that some individuals may have undergone resection for other reasons (19).

Bennet *et al.* reported that 40.3% of patients required surgical intervention due to abdominal symptoms by the third year of follow-up. However, the study did not distinguish between symptomatic and asymptomatic patients at the time of diagnosis, raising the possibility that some individuals in the non-operative management group may have already exhibited symptoms requiring surgery at baseline (24). Notably, only 56.5% of patients received SSAs, compared to nearly 95% in the Daskalakis study (19) and 100% in the current analysis. Given the well-established antitumor effects of SSAs, such treatment may have contributed to reducing tumor and MLNM progression, thereby lowering the risk of symptom development. The results indicate that PRRT treatment may also have a measurable impact on MLNM size, as a greater volume reduction was observed in the lutetium-treated group compared to non-treated patients. This contrasts with the Blažević *et al.* analysis (25), where only 3.8% of patients demonstrated a size response in MLNM. Importantly, none of the PRRT-treated patients in the present study developed symptoms during follow-up.

The observed negative median volume change and TGR support a conservative management strategy, particularly in the context of effective medical therapies. These findings are consistent with those of Blazevic *et al.*, who reported that only 13.5% of mesenteric metastases larger than 1 cm exhibited TG, albeit using RECIST 1.1 criteria as their measurement methodology (25). The one-dimensional assessment applied in the RECIST criteria may not adequately reflect changes in total volume or lesion morphology, potentially resulting in an underestimation or misrepresentation of tumor size, particularly in irregularly shaped lesions. In contrast, the current study utilized a three-dimensional volumetric assessment combined with TGR analysis, providing a potentially more accurate estimation of tumor burden and deeper insights into tumor behavior. A key advantage of TGR is its incorporation of the time interval between imaging assessments, unlike RECIST 1.1, allowing for a dynamic evaluation of tumor progression.

MLNM levels II and III down were the most common locations in patients with symptoms, both at diagnosis and among those who later developed symptoms, suggesting a stronger association with clinical manifestation. However, as abdominal symptoms were observed across various MLNM classification levels, no single location could be definitively associated with symptom onset.

Although no regular control group was available, the 5-year and 10-year OS rates were comparable to those reported in a previous study (19). However, the small sample size limits the strength of these findings and definitive conclusions regarding this outcome cannot be fully assessed.

Several limitations of this study should be acknowledged. The retrospective design inherently carries a risk of selection and information bias. The sample size of 44 and 54 patients, respectively, may not provide sufficient statistical power to detect subtle differences in outcomes and limit the generalizability of the results.

Given the study’s focus, incorporating a randomized control group for prophylactic surgery would not have been appropriate, as this would have limited the assessment of symptom development and natural tumor and MLNM behavior over time. Instead, the study design was intended to provide insights into the outcomes of a non-operative approach. The heterogeneity of treatment modalities, including the use of SSAs and PRRT, may also introduce confounding variables, making it difficult to isolate the effects of medical management alone.

Furthermore, the study utilized a three-dimensional measurement technique for assessing MLNM volume change and TGR. This method provides a more accurate representation of tumor size than one-dimensional RECIST criteria, particularly when assessing a single lesion. However, as TGR calculations rely on volumetric measurements, irregularly shaped lesions may introduce variability in volume estimation, potentially impacting the accuracy of derived TGR values. This risk was minimized by having a senior radiologist review all CT scans.

Although conducted at a single tertiary care center, the findings may be applicable to other institutions with expertise in managing stage IV SI-NETs. Nevertheless, variations in access to advanced therapies could affect the generalizability of the results. Given the rarity of this disease, future prospective multicenter studies with standardized treatment and follow-up protocols are needed to validate these findings in larger patient cohorts.

A non-operative approach in initially asymptomatic patients with stage IV SI-NETs was associated with a low incidence of symptom development requiring surgery, while MLNM demonstrated only modest size progression. These findings suggest that prophylactic palliative resection may not be justified in these patients, supporting a strategy of selective surgery based on clinical indication. Our results indicate that approximately 90% of such patients could be exposed to unnecessary surgery, with associated morbidity and mortality if a prophylactic approach were routinely employed. In addition, nearly 20% of those initially operated would require subsequent re-intervention due to complications such as hernias and adhesions.

## Declaration of interest

The authors declare that there is no conflict of interest that could be perceived as prejudicing the impartiality of the work reported.

## Funding

This study was supported by grants from the Swedish Cancer Foundation, the Bergholms Foundation, the Erikssons Foundation, the Lions Cancer Fund, and the Ihre Foundation. The sponsors had no role in the collection, analysis or interpretation of data. The research was not registered in an independent, institutional registry.
